# Event‐related fMRI at 7T reveals overlapping cortical representations for adjacent fingertips in S1 of individual subjects

**DOI:** 10.1002/hbm.22310

**Published:** 2013-09-03

**Authors:** Julien Besle, Rosa‐Maria Sánchez‐Panchuelo, Richard Bowtell, Susan Francis, Denis Schluppeck

**Affiliations:** ^1^ Visual Neuroscience Group, School of Psychology, University of Nottingham NG7 2RD Nottingham United Kingdom; ^2^ Sir Peter Mansfield Magnetic Resonance Centre, School of Physics and Astronomy, University of Nottingham NG7 2RD Nottingham United Kingdom

**Keywords:** High‐resolution functional MRI, Human, Somatosensory cortex, Tactile Perception

## Abstract

Recent fMRI studies of the human primary somatosensory cortex have been able to differentiate the cortical representations of different fingertips at a single‐subject level. These studies did not, however, investigate the *expected overlap* in cortical activation due to the stimulation of different fingers. Here, we used an event‐related design in six subjects at 7 Tesla to explore the overlap in cortical responses elicited in S1 by vibrotactile stimulation of the five fingertips. We found that all parts of S1 show some degree of spatial overlap between the cortical representations of adjacent or even nonadjacent fingertips. In S1, the posterior bank of the central sulcus showed less overlap than regions in the post‐central gyrus, which responded to up to five fingertips. The functional properties of these two areas are consistent with the known layout of cytoarchitectonically defined subareas, and we speculate that they correspond to subarea 3b (S1 proper) and subarea 1, respectively. In contrast with previous fMRI studies, however, we did not observe discrete activation clusters that could unequivocally be attributed to different subareas of S1. Venous maps based on T2*‐weighted structural images suggest that the observed overlap is not driven by extra‐vascular contributions from large veins. *Hum Brain Mapp 35:2027–2043, 2014*. © **2013 The Authors Human Brain Mapping published by Wiley Periodicals, Inc.**

## INTRODUCTION

The ability to measure reliable and detailed cortical maps in individual subjects is a key feature in applying fMRI to clinical settings. Mapping the cortical representation of the fingertips of the hand could be especially important for understanding and characterizing abnormal sensory‐motor cortical functions or cortical reorganization, for example, following disruption of normal developmental periods, traumatic brain injury, stroke, peripheral nerve damage [Tecchio et al., 2002] or dystonia [Hinkley et al., 2009]. Recent fMRI studies at high (3T) [Nelson and Chen, 2008; Schweizer et al., 2008] or ultra high (7T) magnetic field [Sanchez‐Panchuelo et al., 2010; Stringer et al., 2011] in normal subjects have shown that it is possible to spatially differentiate activations following the stimulation of adjacent fingertips in the human primary sensory cortex (S1).

In nonhuman primates, and also likely in humans, S1 is subdivided into at least four functionally and cyto‐architectonically distinct subregions (in antero‐posterior order: areas 3a, 3b, 1 and 2), that each contains a complete representation of the hand and fingertips [Geyer et al., 1999; Merzenich et al., 1987; Pons et al., 1987; Sanchez‐Panchuelo et al., 2012]. Importantly, the average cell receptive field size in nonhuman primates has been found to increase from area 3b to area 2 [Hyvärinen and Poranen, 1978], being mostly restricted to one finger in area 3b [Iwamura et al., 1983a; Pons et al., 1987] but often extending over several fingers in areas 1 and 2 [Iwamura et al., 1983b, 1985]. This leads to the hypothesis that noninvasive neuroimaging methods recording the aggregate activity of cell populations should reveal spatially overlapping responses to adjacent fingertips in areas 1 and 2, but not in area 3b. In contrast, scalp EEG [Gandevia et al., 1983], intracranial EEG [Hsieh et al., 1995] and MEG [Biermann et al., 1998; Hoechstetter et al., 2001] studies have shown that early somatosensory potentials/fields are suppressed for simultaneous, compared with separate, fingertip stimulation for responses believed to be generated in areas 3b and 1, suggesting representational overlap in both of these subregions. Such suppressive effects, however, are only indirect evidence of overlap and could be explained by lateral inhibition rather than overlapping representations. fMRI provides better spatial resolution than EEG/MEG, and so should allow a more direct measure of spatial overlap between fingers. FMRI studies at 1.5 T have shown large activation overlaps [Krause et al., 2001; Kurth et al., 2000] and suppressive effects [Ruben et al., 2006] between adjacent fingers in cortical locations compatible with areas 3b, 1 and 2, with an increase in overlap from anterior to posterior regions. However, these studies may have over‐estimated the extent of cortical overlap as the blood oxygenation level dependent (BOLD) effect underlying fMRI is only an indirect measure of neuronal activity and imposes a spatial blur that increases the spatial extent of responses [Engel et al., 1997]. In particular, at 1.5T the origin of the BOLD effect is thought to be dominated by intravascular large vessel effects, rather than signal from the cortical microvasculature [Boxerman et al., 1995]. Higher magnetic field strength provides an increased BOLD contrast [Van der Zwaag et al., 2009], a reduction in the relative contribution of physiological noise thanks to reduced voxel size [Triantafyllou et al., 2005], and reduces the intravascular contribution from draining veins [Duong et al., 2003; Yacoub et al., 2001]. This higher spatial resolution and specificity should allow more accurate mapping of overlapping representations in different subregions of S1 in individual subjects.

Recent fMRI single‐subject mapping studies of S1 at 3T or 7T have focussed on resolving the representations of adjacent fingers, rather than estimating the spatial overlap. Some studies have used differential designs [Sanchez‐Panchuelo et al., 2010] or differential contrasts [Stringer et al., 2011] that reduce the detectability of overlapping activity due to overlapping receptive fields [Dumoulin and Wandell, 2008]. Other studies have used conservative statistical thresholds in order to elicit small and spatially separate activation clusters to define the locus of individual fingers [Nelson and Chen, 2008; Stringer et al., 2011], whereas others may not have had sufficient statistical power to elicit overlapping clusters because of inherently low SNR [Schweizer et al., 2008]. In addition, in some of these studies the number of voxels entering multiple testing control procedures was not limited, potentially leading to undesirably conservative corrected thresholds [Nelson and Chen, 2008; Stringer et al., 2011].

The aim of our study is to use ultra‐high magnetic field (7T) to acquire high spatial resolution fMRI data (1.5 mm isotropic voxels), and use an event‐related (ER) design in which we stimulate the five fingertips of the left‐hand independently, to assess the overlap of cortical representation of fingertips in S1 in individual subjects. Using a phase‐encoding design as a functional localizer [Sanchez‐Panchuelo et al., 2010], we restrict our analysis of the ER data to voxels in the vicinity of S1. In conjunction with the improved SNR afforded by ultra‐high magnetic field, this small‐volume correction allows us to demonstrate overlapping BOLD responses to stimulation of adjacent and nonadjacent fingertips. We also demonstrate that anterior regions of S1, likely to correspond to area 3b in the monkey, show less overlap (more fingertip specificity) than the posterior region corresponding to areas 1/2, and that this pattern cannot be explained by extra‐cortical vascular contributions. In contrast to previous studies, and despite improved statistical power, using this paradigm and analysis procedure we did not observe discrete activation clusters that could unequivocally be attributed to different subareas of S1.

## MATERIAL AND METHODS

### Subjects

Six right‐handed subjects experienced in fMRI experiments participated in this study (aged 24–36 years, two females). Approval for the study was obtained from the University Ethics Committee and all subjects gave full written consent. Each subject participated in at least two scanning sessions: one functional session at 7T, and one structural session at 3T. The latter was used to obtain a T_1_‐weighted image of the whole brain for image segmentation and cortical unfolding.

### Stimuli and Task

The fingertips of each subjects' left hand were stimulated using five independently controlled, piezo‐electric devices to deliver supra‐threshold vibrotactile stimuli (50 Hz) to ∼1 mm^2^ of the skin (Dancer Design, UK, http://www.dancerdesign.co.uk).

The location of S1 was determined using two to four runs of a phase‐encoding localizer, details of which have been published elsewhere [Sanchez‐Panchuelo et al., 2010]. Following the localizer, six to eight runs of the ER design were carried out. Each run comprised 30 stimulation trials (6 per fingertip), with each trial consisting of two, 0.4 s stimulations at the same fingertip separated by a 0.1 s gap. The onset of each trial was synchronized to the start of an MRI volume acquisition, with a random intertrial interval ranging from 4 to 12 s (in 2 s steps). In each run, trials were randomized such that each fingertip trial was presented exactly once in each successive block of five trials. Trial order within these blocks of five trials was randomized. To maintain subjects' attentional state, subjects performed a two‐interval forced choice discrimination task, alternating between runs over either a visual fixation point dimming task [choosing which interval had lower luminance, Gardner et al., 2008] or a tactile task (choosing in which interval there was a slight increase in stimulus amplitude), with an equal number of runs for each attentional task in a scan session. Subjects reported their response by pressing one of two buttons with their nonstimulated, right hand. No significant interaction was found between attentional task and finger stimulation in five out of six subjects, supporting that this relatively coarse attentional manipulation had no appreciable differential effect in S1 on mapping the digits, and so visual and somatosensory attentional tasks were combined in the ER analysis.

### Image Acquisition

MR data were collected on a 7T system (Philips Achieva) using a volume transmit coil and a 16‐channel receive coil (Nova Medical, Wilmington, MA). To minimize head motion, participants' heads were stabilized with a customized MR‐compatible vacuum pillow (B.u.W. Schmidt, Garbsen, Germany] and foam padding.

Functional data were acquired using T_2_*‐weighted, multislice, single‐shot gradient echo, echo‐planar imaging (EPI) with the following parameters: TE = 25 ms, SENSE reduction factor 3 in the right‐left (RL) direction, flip angle (FA) = 75°, TR = 2000 ms. The spatial resolution was 1.5 mm isotropic with a field of view (FOV) of 156 × 192 × 42 mm^3^ in right‐left (RL), anterior–posterior (AP) and foot‐head (FH) directions, respectively. Magnetic field inhomogeneity was minimized using an image‐based shimming approach [Poole and Bowtell, 2008; Wilson et al., 2002] as described in detail in Sanchez‐Panchuelo et al. (2010).

**Figure 1:**
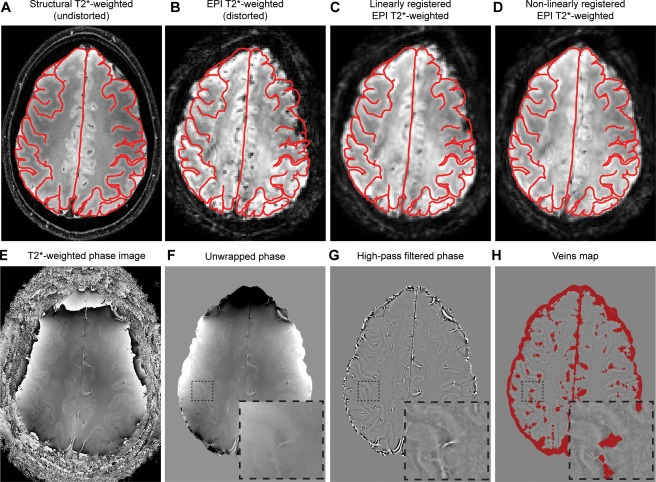
Registration and identification of veins using T2*‐weighted magnitude and phase images. **A**: Example T2*‐weighted anatomical image with same slice prescription and coverage as functional data (**B**,**C**,**D**), but improved in‐plane spatial resolution (0.25 × 0.25 mm^2^). Note that the anatomical images are not subject to the same geometric distortions as the functional EPI images. B: Mean EPI (T_2_*‐weighted) functional image with no registration applied, highlighting large regions of mismatch due to geometric distortions; C: after affine registration (FSL/FLIRT), which improves correspondence; D: following non‐rigid registration of mean functional image to anatomical image using FNIRT, showing a close match. Red line, trace of gray matter cortical surface from high‐resolution T_2_*‐weighted anatomical image. **E**. Example T2*‐weighted phase image (same subject, slice and resolution as A). Phase images are unwrapped (**F**) and high‐pass filtered (**G**) in order to emphasize abrupt phase changes corresponding to changes in magnetic susceptibility in and around veins. **H**: A map of veins is approximated by thresholding the unwrapped, filtered phase image and convolving the identified voxels with a 2 mm kernel. [Color figure can be viewed in the online issue, which is available at http://wileyonlinelibrary.com.]

The functional runs were followed by the acquisition of high‐resolution, T_2_^*^‐weighted axial images (0.25 × 0.25 × 1.5 mm^3^ resolution; TE/TR = 9.3/457 ms, FA = 32°, SENSE factor = 2) with the same slice prescription and coverage as the functional data. Large veins could be identified from these T_2_^*^‐weighted images, (see Fig. 1E–H). To assess the tissue specificity of the activation measured from the functional data, venous vessel masks were generated from the phase of the high resolution, T_2_^*^‐weighted images [Harmer et al., 2012]. A high‐resolution 3D MPRAGE dataset (1 mm isotropic resolution, linear phase encoding order, TE/TR 3.7/8.13 ms, FA = 8°, TI = 960 ms) was collected at 3T. These data were acquired at 3T for ease of segmentation prior to flattening, as images acquired at 3T display less B_1_‐inhomogeneity‐related intensity variation than 7T data. The T_2_^*^‐weighted images were also used for registration with these whole‐head anatomical T_1_‐weighted images acquired at 3T.

### Data Analysis

Preprocessing steps were carried out using tools in FSL [Smith et al., 2004], and statistical analysis of functional imaging data was performed using mrTools (http://www.cns.nyu.edu/heegerlab) in Matlab (The Mathworks, Natick, MA).

#### Preprocessing

The functional data were aligned (motion‐corrected) to the last volume of the functional data set acquired closest in time to the high‐resolution T_2_*‐weighted volume (the reference EPI volume). To account for scanner drift and other low‐frequency signals, all time‐series were high‐pass filtered (0.01 Hz cut‐off) and data were then converted to percent‐signal change for subsequent statistical analysis.

#### Statistical Analysis

##### Phase‐encoding localizer and region of interest (ROI) definition

The phase/amplitude and coherence of the best fitting sinusoid at the stimulation frequency were estimated for each run using standard methods [Sanchez‐Panchuelo et al., 2010]. Regions of Interest (ROI) corresponding to each fingertip were formed by dividing phase values into five bins of 2π/5 width (spanning phase values from 0 to 2π) and selecting spatially contiguous regions of voxels from the same bin, which were above an empirically determined coherence threshold of 0.25 (equivalent to an uncorrected *P*‐value of 0.006). To overcome the issue of the HRF delay, we averaged time series from forward and reverse order phase encoding scans, as is routinely performed in retinotopic mapping. Note that the phase value of a voxel, and hence, the assignment to a preferred digit, is independent of the coherence value. ROI selection was restricted to voxels within the cortical gray matter by first transforming the phase map into the structural whole‐head space using nonlinear transformation coefficients (see below), and then converting the ROI voxel coordinates back into the functional volume space. These fingertip ROIs were subsequently used as independent ROIs [Kriegeskorte et al., 2009] in the analysis of the event‐related data to (1) limit the number of multiple comparisons and (2) allow group‐level inference tests to be conducted.

To allow comparison to activation patterns published previously, we also report the distance between the centers‐of‐mass of fingertip ROIs by measuring both the Euclidian distance (which is likely to underestimate the cortical distance) and the Dijkstra distance [Dijkstra, 1959] on the cortical surface mesh, which respects the geometry of the cortical folding pattern. The Dijkstra distance was computed as the length of the shortest connected path between vertices closest to the centers‐of‐mass.

##### Event‐related (ER) design

The event‐related data were fitted using two variations of the general linear model (GLM). The first used a fixed canonical hemodynamic response function (HRF) model in order to estimate a single response magnitude parameter per fingertip stimulation condition. The second used a HRF deconvolution model to estimate the shape of the HRF in response to each fingertip stimulation condition.

##### Canonical HRF model

In this GLM, each fingertip stimulation was modeled as a 1 s boxcar and convolved with a canonical double‐gamma HRF and its orthogonalized temporal derivative, resulting in 10 regressors (2 regressors per fingertip stimulation condition). The GLM was fitted in a two step‐process to allow for intersubject variation in hemodynamic delay. In the first step, we fixed the timing parameters of the canonical double‐gamma HRF (time‐to‐peak for the positive and negative gamma functions, 6 s and 16 s, respectively) and fitted the GLM using ordinary least squares. We then estimated in each phase‐encoding‐defined fingertip ROI the time to the positive and negative peaks of the estimated HRF (double‐gamma + derivative) for the corresponding fingertip condition. For each subject, we averaged these estimates across the five fingertip ROIs (see Table 1) and used them as timing parameters of the gamma functions in the second step. In this second step the GLM was fitted using generalized least‐squares in order to correct for temporal correlation of the noise [Burock and Dale, 2000; Wicker and Fonlupt, 2003]. The noise correlation matrix was estimated at each voxel using Tukey tapers [Woolrich et al., 2001] from the time‐series averaged across within‐slice, square regions of 20·20 voxels, excluding voxels outside the brain.

**Table 1:** Time to the maximum positive peak and negative undershoot of the estimated HRF in the canonical GLM analysis, and number of voxels analysed for each subject

	HRF parameters		Voxels analyzed
	Positive peak (s)	Negative peak (s)	
Subject 1	3.78	11.61	5215
Subject 2	3.63	12.79	7460
Subject 3	5.63	16.41	7282
Subject 4	3.91	14.09	8425
Subject 5	3.08	9.18	9301
Subject 6	4.88	15.13	6864
Average	4.2	13.2	7424

For each fingertip condition, we tested on a voxel‐by‐voxel basis whether the magnitude parameter estimate was greater than 0 using a one‐sided *T*‐test taking into account the noise correlation matrix to reduce bias [Burock and Dale, 2000; Wicker and Fonlupt, 2003; Woolrich et al., 2001]. In order to reduce the number of inference tests, the analysis was restricted to voxels in the vicinity of the S1 ROIs identified using the phase‐encoding localizer. This S1 ROI was expanded to be within 5 voxels of any fingertip ROI (excluding voxels outside the brain) to account for the fact that the phase‐encoding paradigm is blind to voxels responding to all five fingertip stimulations. The average number of voxels analyzed per subject was 7425 ± 624 (SEM), equivalent to a volume of 25 ± 2.1 cm^3^ (see Table 1).

Family‐wise error (FWE) correction was performed across voxels using a step‐down method [Holm, 1979] after estimating the number of true null hypotheses using a least‐squares method [Benjamini et al., 2006; Hsueh et al., 2003]. Since FWE‐correction methods can be unduly conservative in neuroimaging, we compared FWE‐adjusted maps with those obtained using false‐discovery rate (FDR) correction [Benjamini and Hochberg, 1995]. FDR correction was performed using an adaptive step‐up method [Benjamini et al., 2006]. All adjusted *P*‐values were converted to quantiles of the standard normal distribution (*Z*‐values).

##### HRF deconvolution model

**Figure 2:**
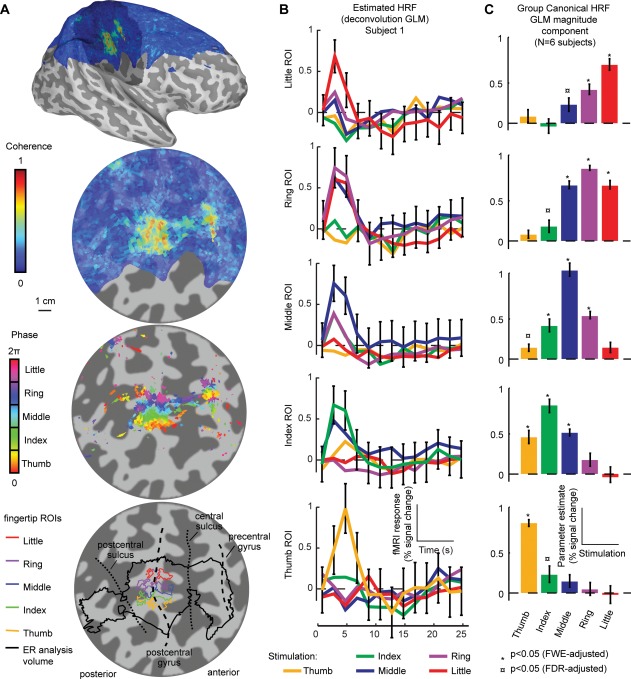
**A**: Localization of S1 and definition of fingertip‐specific ROIs using the phase‐encoding paradigm (data from subject 1). Coherence maps, phase maps and ROIs are displayed on inflated 3D model of the right hemisphere cortical surface (top) and flattened cortical patch (bottom 3 maps). Dark gray, areas of negative curvature (sulci); light gray, areas of positive curvature (gyri); shaded area on the 3D model, location of the cortical flat patch. The scale was estimated from the length along the cortical surface of a straight line drawn on the flattened patch. Coherence values are the maximum intensity projection of coherence across coordinates corresponding to different cortical depths. Note that not all surface points of the patch have an associated value, because of the partial FOV of the functional images as shown in blue. Phase maps for the corresponding dataset are thresholded at a coherence value of 0.25. Phase values (in radians) and corresponding preferred stimulus location (fingertip) are shown. Phase values are displayed at a relative cortical depth of 0.8 (where 0 is the white/gray matter interface and 1.0 the pial surface). Fingertip‐specific ROIs (bottom) are defined as all contiguous voxels within a given phase‐interval, over a coherence threshold of 0.25 and within the cortical sheet. The black outline indicates the volume in which the event‐related analysis was performed to limit the number of multiple comparisons (constructed by expanding the 5 fingertip ROIs by 5 voxels in 3D space). **B**: Estimated HRF from the deconvolution GLM for each stimulation condition for subject 1, averaged across voxels of each ROI. Error bars, voxel‐wise parameter standard errors averaged across voxels of each ROI (preferred fingertip condition only). C: Parameter estimates of the magnitude component from canonical HRF GLM fit for each stimulation condition, averaged across voxels of each ROI and across all subjects (*N* = 6). Error bars, standard error of the ROI‐averaged parameters across subjects. The two‐way interaction between ROIs and fingertip stimulation was highly significant (see text). Symbols, statistical significance of each factor combination compared to zero, adjusted for multiple testing across all combinations (see Material and Methods section). [Color figure can be viewed in the online issue, which is available at http://wileyonlinelibrary.com.]

To estimate the shape of HRFs generated by stimulation of different fingertips (Fig. 2B) a “deconvolution” GLM was fitted to the data [Dale, 1999; Gardner et al., 2005]. In this model, each fingertip HRF model comprised 13 time‐shifted regressors that each modeled the response at a given delay after the stimulation (0–12 TRs). This GLM was fitted using ordinary least‐squares and resulted in 13 parameter estimates per fingertip stimulation condition, representing the estimated shape and magnitude of the HRF.

##### Functional division of fingertip‐specific ROIs

Fingertip ROIs for each digit were divided based on the degree of activation overlap between fingertip stimulation conditions. First, we superimposed the voxel‐wise magnitude estimates from the canonical HRF GLM analysis for each fingertip stimulation condition on the flat map (see next section). Next, we found the boundary (line on the cortical surface) separating voxels responding to the stimulation of only one or two adjacent fingertips from those voxels responding to more than two fingertip stimulation conditions. This boundary was then projected at all cortical depths, and used as the division between anterior and posterior ROIs.

##### Assessing specificity of fingertip responses

To quantify group‐level effects, parameter estimates of the canonical HRF GLM analysis were averaged across voxels in the phase‐encoding ROIs for each fingertip for each subject. This gave single parameter estimates per ROI, per fingertip stimulation condition, and per subject that were then used to plot group averages and perform ANOVA tests. For group measures, parameter estimate averages were normalized to eliminate variability due to differences in CNR between subjects (dividing by the maximum of each subject's value and multiplying by the average maximum value across subjects). A two‐factor ANOVA was performed to test the interaction between the ROI and the fingertip that was stimulated. Second, having divided ROIs into anterior and posterior sections based on overlap, a three‐factor ANOVA was performed, adding anatomical location (posterior/anterior) as an additional factor. Homoscedasticity was not assumed and *P*‐values were corrected using Greenhouse‐Geisser degrees of freedom correction. In addition, each ROI‐averaged parameter estimate was compared to 0 using *T*‐tests, and *P*‐values corrected for multiple comparisons using Hommel's method [Hommel, 1986].

Additional quantitative analyses were also performed to estimate: the overlap of fingertips; the proportion of voxels maximally responding to each fingertip stimulation for a range of thresholds; the overlap ratio [Krause et al., 2001; Kurth et al., 2000; Ruben et al., 2006], which is reported at FDR *P* < 0.05.

#### Alignment and projection to surfaces and flattened patche

Automated cortical segmentation of the anatomical images and reconstruction of the white/gray and gray/pial cortical surfaces were performed using Freesurfer [http://surfer.nmr.mgh.harvard.edu/; Dale et al. 1999]. The mrFlatMesh algorithm (VISTA software, http://white.stanford.edu/software/) was used to create flattened representations of the cortical regions surrounding the central sulcus and post‐central gyrus of the right hemisphere.

In order to render the results on surface and flattened representations, statistical maps were moved from functional data acquisition space into anatomical space, in which cortical surface reconstruction had been performed. First, we estimated the linear alignment matrix between the undistorted, partial FOV, high‐resolution T_2_*‐weighted volume and the T_1_‐weighted whole‐head anatomical images using an iterative, multi‐resolution robust estimation method [Nestares and Heeger, 2000]. Second, we estimated the alignment between the (distorted) reference EPI frame (see Preprocessing section) and the undistorted, partial FOV, high‐resolution T_2_*‐weighted volume. Ultra‐high field MRI is susceptible to relatively large field inhomogeneities that can cause significant geometric distortions in EPI data [Poole and Bowtell, 2008]. Even though image‐based shimming was used here to minimize such inhomogeneities, residual distortions will remain, prohibiting the use of a rigid‐body registration between functional EPI data and anatomical images (Fig. 1A–D). To address this problem, nonlinear alignment estimation was performed using FSL's nonlinear registration algorithm [FNIRT, Andersson et al., 2007]. Note that ROI definition depended on this nonlinear registration because it was restricted to the cortical surface. All other analyses were performed in the space of the original functional data (after motion correction) and only the resulting statistical maps were linearly and nonlinearly transformed for display on the cortical surface.

To project the statistical maps onto the 3D reconstruction of the cortical surface we applied the aforementioned alignments: functional maps were first nonlinearly transformed into the space of the structural T2* volume using FSL FNIRT's *applywarp* and then linearly transformed from the structural T2* to the whole‐head volume space. Statistical values were sampled (using nearest‐neighbour interpolation) at the coordinates of the inner (white/gray) and outer (gray/pial) surfaces and at 9 intermediate, equally spaced cortical depths. Values at a given coordinate (and cortical depth) in the original whole‐head anatomical space were then displayed on the inflated surface or flattened patch using the known direct correspondence between each point of the original inner/upper surface and each point of the inflated/flattened surface.

For coherence values from the phase‐encoding analysis and parameter estimate maps from the ER GLM analysis, the values displayed correspond to the maximum intensity projection of the relevant surface points across the 11 cortical depths. For phase maps in the phase‐encoding data, values were computed as the phase of the complex average across cortical depths (using amplitude and phase values at each depth).

To convey the size and statistical significance of parameters generated using the canonical HRF GLM analysis, parameter estimates were color‐coded and each pixel's transparency alpha value set to reflect the *Z*‐value in the corresponding inference test. When superimposing two fingertip activation maps, colours were additively combined according to the formula: *C* = *C*_A_*(1−*C*_B_) + *C*_B_, where *C*_A_ and *C*_B_ are RGB vectors between 0 and 1, premultiplied by their respective alpha values. When superimposing more than two maps, the formula was applied iteratively, effectively resulting in a whitish hue for any voxels that were activated by more than two conditions.

## RESULTS

### Overlap of BOLD Responses for Different Fingertips

To study the overlap of activation due to stimulation of the different fingertips, we estimated the event‐related BOLD response to each fingertip stimulation in nonoverlapping fingertip ROIs defined from the phase‐encoding localizer. (Fig. 2A, shown for Subject 1). We found areas with high coherence values (responding preferentially to a given fingertip) in the posterior bank of the central sulcus of the contralateral (right) hemisphere, extending posteriorly on the post‐central gyrus. The corresponding phase map was organized along an inferior/superior, lateral‐medial axis from low phase values (preference to thumb) to high phase values (preference to little finger). Table 2 summarizes the number of voxels in each finger‐specific ROI and Table 3 summarizes average distance between ROIs.

**Table 2:** Number of voxels per ROI and for each subject. Each voxel corresponds to 3.375µl of cortical tissue

	Number of voxels in fingertip ROIs				
	Thumb	Index	Middle	Ring	Little
Subject 1	128	103	85	131	51
Subject 2	180	95	93	203	46
Subject 3	226	58	58	88	40
Subject 4	387	72	79	170	224
Subject 5	219	162	32	361	49
Subject 6	159	84	65	143	70
Average	216	9**6**	6**9**	18**3**	80

**Table 3:** Average distance between centers‐of‐mass of anterior (upper triangle) and posterior (lower triangle) fingertip phase‐encoding‐defined ROIs (in mm ± standard error across subjects)

Surface (Dijkstra) distance between ROI centres‐of‐mass						
	D1	D2	D3	D4	D5	
D1		6.1 ± 0.8	10.5 ± 0.8	14.9 ± 0.6	20.2 ± 0.6	Anterior ROIs
D2	6.4 ± 1.3		5.9 ± 0.8	10.5 ± 1.3	15.8 ± 1.3	
D3	11.3 ± 0.9	5.6 ± 1.5		5.5 ± 0.5	10.2 ± 0.6	
D4	15.6 ± 0.8	10.7 ± 1.4	6.4 ± 0.5		5.5 ± 0.5	
D5	20.0 ± 0.6	14.8 ± 1.8	9.7 ± 0.6	5.7 ± 1.2
	Posterior ROIs					

Euclidian distance between ROI centers‐of‐mass						
	D1	D2	D3	D4	D5	
D1		5.0 ± 0.8	9.0 ± 0.8	12.0 ± 0.8	15.4 ± 0.8	Anterior ROIs
D2	5.2 ± 0.9		5.4 ± 0.3	8.9 ± 0.8	12.6 ± 0.7	
D3	8.4 ± 0.9	5.0 ± 1.1		4.6 ± 0.3	8.5 ± 0.4	
D4	12.5 ± 1.0	9.9 ± 1.1	5.5 ± 0.3		4.7 ± 0.4	
D5	15.6 ± 1.4	13.0 ± 1.5	8.4 ± 0.7	4.1 ± 0.8		
	Posterior ROIs					

Distances between ROIs were measured on the surface mesh for each subject (surface distance) to give a realistic estimate of separation on the cortical surface; Euclidian distances (in 3D, not respecting the geometry of the cortical sheet) are included to allow comparison with previously published results.

Figure 2B plots the estimated ER BOLD responses evoked by the five different fingertip stimulations in each fingertip‐specific ROI for Subject 1. As expected, the BOLD response in a given fingertip ROI was largest for the corresponding fingertip stimulation. Interestingly, most ROIs also showed positive responses for adjacent fingertip stimulations. Figure 2C plots the magnitude parameter estimates of the fixed HRF GLM model for each fingertip stimulation condition in each ROI averaged across subjects, confirming this pattern of maximum magnitude estimates for the fingertip stimulation corresponding to the ROI, and significantly larger than baseline responses for up to four adjacent fingertip stimulation conditions. The interaction between fingertip ROI and stimulation condition was highly significant (*F*_16,80_ = 71.7, *P* < 10^−16^, Greenhouse‐Geisser correction: *F*_1,5_ = 71.7, *P* < 10^−3^).

**Figure 3:**
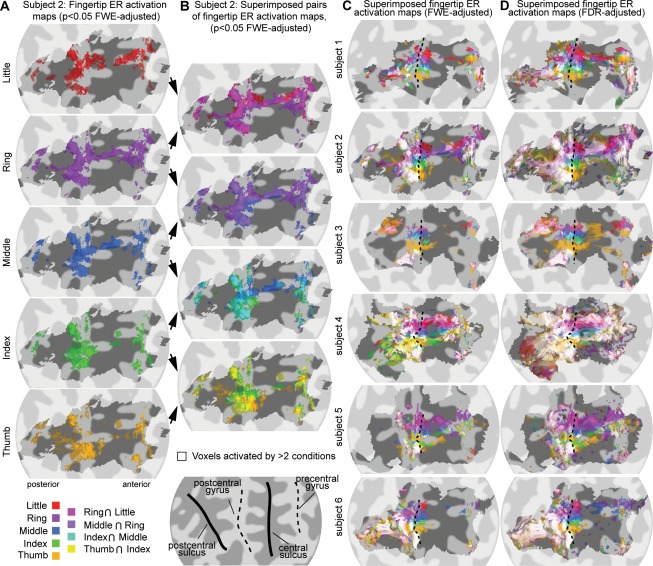
Maps of overlapping activation for event‐related data. **A**. Parameter estimate maps for the magnitude component of each stimulation condition in the canonical HRF GLM analysis for subject 2, displayed on a flattened cortical patch. Saturation of each colour map represents the amplitude of the parameter estimate. Transparency represents the corresponding statistical significance (thresholded at *P*‐value < 0.05, FWE‐adjusted). Shaded area, voxels included in the analysis. **B**. Parameter estimate maps for adjacent fingers (subject 2, *P* < 0.05 FWE‐adjusted) superimposed using additive colour blending scheme described in Material and Methods. Voxels activated by adjacent finger stimulation conditions are shown in intermediate colours (see colour legend). **C**. Parameter estimate maps for all five fingertips superimposed using additive colour blending (*P* < 0.05 FWE‐adjusted), each row shows data from one subject. Colours identical to panel B, but voxels activated by more than two fingertips are shown in de‐saturated (whiter) shades. Second row shows subject 2 as shown in panels A and B. Dashed line indicates a change in the specificity of voxels: voxels anterior to the line show a higher degree of specificity (less overlap). **D**. As for C, but with the less conservative threshold FDR‐adjusted *P* < 0.05. A higher degree of specificity can still be seen in the posterior bank of the central sulcus. [Color figure can be viewed in the online issue, which is available at http://wileyonlinelibrary.com.]

Figure 3A shows parameter estimate maps for each stimulation condition for an example subject, weighted by the corresponding statistical comparison to 0 (FWE‐corrected threshold of 0.05), and shows a complex spatial pattern of response that was not restricted to the simple cortical bands expected from the phase‐encoding maps. Figure 3B shows the overlap of activation for adjacent fingertip stimulation conditions, indicating that the majority of voxels activated by a given fingertip stimulation also significantly respond to neighbouring fingers. The clear exception to this was an area in the posterior bank of the central sulcus, just anterior to the post‐central gyrus, in which most voxels responded only to the stimulation of one fingertip.

On superimposing all five fingertip maps (Fig. 3C, second row), it can be seen that responses in the posterior bank of the central sulcus were generally specific to stimulation of a single fingertip and ordered from thumb to little finger in the inferior/superior direction, corresponding to the somatotopy that is evident in the phase‐encoding maps. However, even in this region, voxels lying between fingertip specific areas often responded to stimulation of the adjacent fingers. In regions posterior to the posterior bank of the central sulcus, most voxels responded to stimulation of more than two different fingertips (white voxels). In regions anterior to the posterior bank of the central sulcus, some voxels showed a specific response, whereas others responded to stimulation of several or all fingertips. Figure 3D shows the superimposed ER activation maps thresholded at a less conservative FDR‐adjusted *P*‐value of 0.05 to increase the number of voxels considered to be responding to several adjacent fingertip stimulations. At this threshold, voxels in the posterior bank of the central sulcus still show some degree of specificity. From the FWE‐adjusted and FDR‐adjusted superimposed ER activation maps it was possible to draw a clear boundary between a region of high fingertip specificity and a region of lower fingertip specificity (dashed line in Fig. 3C, D, see Material and Methods section).

### Spatial Variation in Overlap of Fingertip BOLD Responses Within S1

**Figure 4:**
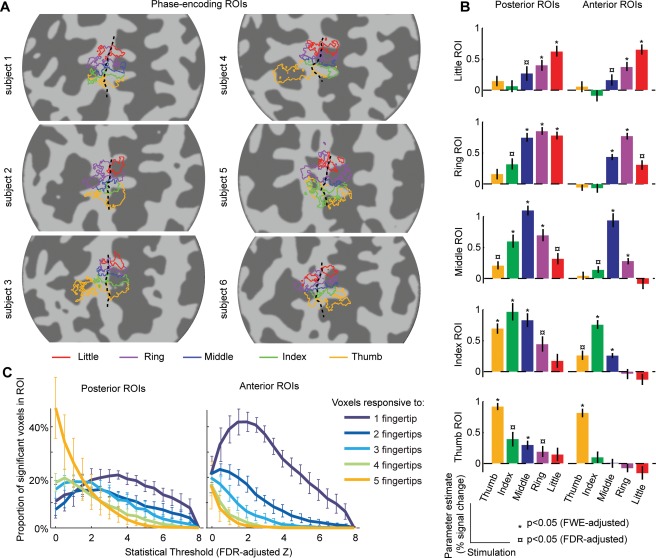
Specificity and activation overlap in the anterior and posterior parts of the ROIs. Note that the anterior part of the ROIs shows a higher degree of specificity. **A:** ROIs defined from the phase‐encoding dataset (see Fig. 2A for details) and divided into anterior and posterior parts based on the event‐related activation map overlap, as illustrated in Figure 3C,D. **B**: Parameter estimates for each of the 5 fingertip stimulation regressors (magnitude component), averaged across voxels in each of the 5 posterior and 5 anterior ROIs defined as in panel A (averaged across subjects). Error bars, standard error across subjects. Other conventions as in Figure 2. The three‐way interaction between fingertip ROI, anterior/posterior ROI and fingertip stimulation was significant. **C**: Proportion of voxels of the phase‐encoding ROIs significantly active in response to only 1, only 2, only 3, only 4 or all fingertip stimulations in the canonical HRF GLM analysis of the ER experiment, plotted as a function of the statistical threshold. Statistical thresholds on the *x*‐axis are expressed as a Z score corresponding to an FDR‐adjusted *P* value. Data averaged across 6 subjects. Error bars, standard error across subjects. The average number of voxels across subjects was 251 ± 13 (SEM) for anterior ROIs and 296 ± 27 for posterior ROIs. [Color figure can be viewed in the online issue, which is available at http://wileyonlinelibrary.com.]

To study differences in overlap/specificity within S1 at the group level, we divided the phase‐encoding ROIs following the boundary drawn in Figure 3C and D. This boundary divides the phase‐encoding ROIs into two roughly equal anterior and posterior halves (Fig. 4A). Group‐averaged ER magnitude parameter estimates, separately averaged within the anterior and posterior ROIs for each fingertip stimulation condition are plotted in Figure 4B. The anterior and posterior ROIs clearly show a differential pattern of interaction, with greater specificity/less overlap for the anterior ROIs. The three‐way interaction between anterior/posterior ROI, fingertip ROI, and fingertip stimulation factors was significant (F_16,80_ = 9.4, *P* = 1.510^−12^, Greenhouse‐Geisser correction: F_1,5_ = 9.4, *P* = 0.028). Even in the anterior ROIs, however, responses to the fingertips adjacent to the preferred fingertip were still significantly different from baseline.

To investigate the extent to which overlapping activations depend on the chosen statistical threshold, Figure 4C shows the proportion of voxels activated in 1, 2, 3, 4 or all fingertip stimulation conditions as a function of the FDR‐adjusted statistical threshold. The anterior and posterior ROIs show clearly different patterns: voxels in anterior ROIs predominantly respond to stimulation of only one fingertip, irrespective of the chosen statistical threshold. In contrast, voxels in posterior ROIs predominantly respond to stimulation of all 5 fingertips at low statistical thresholds, whilst voxels responding to only one fingertip become predominant only at high statistical thresholds.

Table 4 presents the overlap ratios for all four pairs of adjacent fingers, computed for a fixed FDR‐adjusted threshold of *P* < 0.05, separately for the anterior and posterior parts of the phase‐encoding‐defined fingertip ROIs, as well as for all cortical voxels included in the analysis that were posterior to the fingertip ROIs. Across pairs of fingers, there was a significant effect of location (anterior ROIs, posterior ROIs or posterior to ROIs; F(2,5) = 28.9, *P* < 10^−4^). This was due to the overlap ratio being significantly smaller in the anterior part of the ROIs than in either the posterior part of the ROI [t(5) = 7.41, *P* < 0.001] or the cortical volume posterior to the ROIs [t(5) = 6.68, *P* < 0.002].

**Table 4:** Average overlap ratios (in percent ± standard error across subject) for adjacent fingertip activations (FDR‐corrected)

	Anterior ROIs	Posterior ROIs	Voxels posterior to ROIs
D1&D2	35.5 ± 8.0	58.1 ± 10.7	65.2 ± 6.8
D2&D3	36.9 ± 6.6	69.8 ± 7.2	69.7 ± 5.2
D3&D4	51.4 ± 5.2	80.3 ± 6.2	74.5 ± 3.4
D4&D5	47.4 ± 6.6	75.1 ± 6.5	72.9 ± 3.2
Average	42.8 ± 5.6	70.8 ± 7.3	70.6 ± 4.2

Ratios are computed as the number of voxels activated by two adjacent fingertips divided the mean number of voxels activated by each fingertip stimulation, in any of the anterior phase‐encoding‐defined ROIs, posterior phase‐encoding ROIs, and in the analysis volume posterior to any fingertip ROI (restricted to the cortical surface).

### Spatial Overlap and Venous Contributions

**Figure 5:**
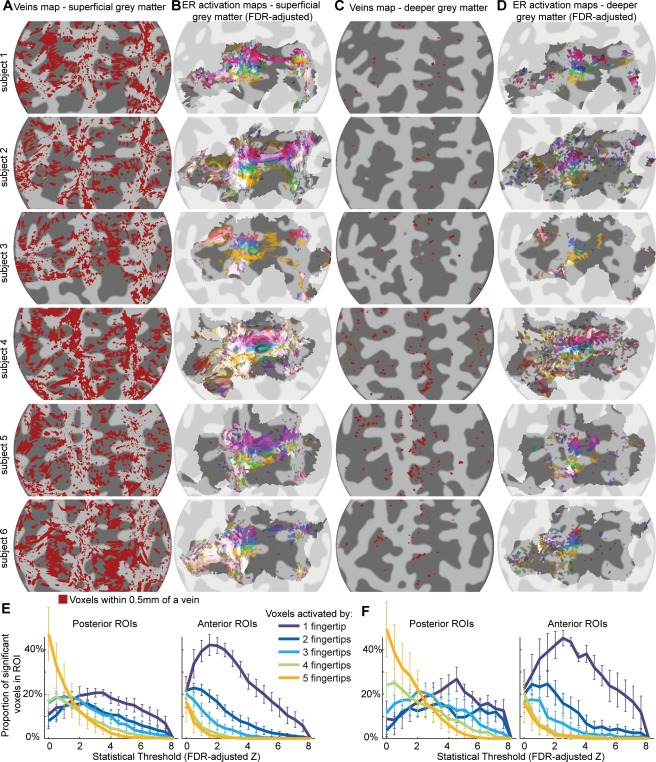
Activation maps at superficial (i.e., near pial surface] and deeper (i.e., near the boundary with white matter] cortical depths and comparison with vein maps. **A**: Map of voxels near the surface of the cortex identified as being within 0.5 mm of a large vein. This flat map corresponds to voxels in the upper 10% of the segmented gray matter. Veins were identified using the T2*‐weighted phase images (see Fig. 1B and Material and Methods]. **B**. Superimposed activation maps for the 5 fingertip stimulation conditions (see Fig. 2 for details] at the same cortical depth as A. **C**. As A for voxels falling in the lower 10% of the segmented gray matter. **D**. As B for voxels falling in the lower 10% of the segmented gray matter. **E**. Percentage of voxels identified as significantly active within the anterior (left) or posterior (right) phase‐encoding ROIs, excluding voxels in the vicinity of large veins, as a function of the statistical threshold (for details, see Fig. 4C]. The average number of such voxels across subjects was 214 ± 7 **(SEM)** for anterior ROIs and 244 ± 23 for posterior ROIs, representing, respectively, 86 and 82% of voxels in these ROIs. **F**. As E, but in this case only including voxels found in the vicinity of large veins. The average number of such voxels was 36 ± 6 for anterior ROIs and 52 ± 8 for posterior ROIs, representing, respectively, 14 and 18% of voxels in these ROIs. [Color figure can be viewed in the online issue, which is available at http://wileyonlinelibrary.com.]

To assess the possibility that the regional differences in specificity might be due to differential effects of large veins, activation maps were compared with vein maps (Fig. 5) formed at different cortical depths. Close to the pial surface, voxels in the vicinity of large veins (see Material and Methods section) were far more numerous (Fig. 5A) than deep in the cortex (Fig. 5 **C**). Correspondingly, more activation was seen close to the cortical surface (Fig. 5 **B**) than close to the white matter border (Fig. 5D), suggesting that at least part of the activation may result from extra‐vascular BOLD contrast due to large veins. However, there was no clear‐cut evidence that this drop of activation with cortical depth was stronger for voxels commonly activated by several fingertips than for those voxels that displayed a response specific to one fingertip, or that veins were more likely to be present over the gyri than the sulci in the region which was studied (the proportion of voxels in the vicinity of large veins did not differ significantly between posterior and anterior ROIs, 17.6 ± 4.4% SEM versus 14 ± 4.2%, respectively, *P* = 0.31. We also plot the proportion of voxels activated by different numbers of fingertips as a function of the statistical threshold (as in Fig. 4C) after excluding voxels labelled as being close to veins (Fig. 5E), or for those voxels classified as veins (Fig. 5F). For both posterior and anterior ROIs, the pattern was very similar to that shown in Figure 4C, suggesting that the responses from regions close to large veins do not explain the regional differences in overlap between the anterior and posterior parts of S1.

## DISCUSSION

We have used an event‐related (ER) design at 7T to locate the cortical regions of S1 responding to the stimulation of different fingertips of the left hand in single subjects. Our aim was specifically to quantify the spatial overlap of responses to the stimulation of different fingertips in individual subjects, in contrast to recent studies which aim to map the location of individual fingertips, for example using a phase‐encoding [Sanchez‐Panchuelo et al., 2010] or a block [Schweizer et al., 2008; Stringer et al., 2011] design. Our results show that there are two distinct regions in S1, an anterior region showing little overlap, and a posterior region showing large overlap of responses (up to all five fingertips).

### Event‐Related Design at 7T Shows Overlapping BOLD Responses to Different Fingertip Stimulations in Single‐Subjects

We quantified the overlap of responses using a number of approaches: (i) by averaging the ER parameter estimates across independently defined ROIs responding preferentially to each of the five different fingertips, (ii) by identifying the proportion of voxels responding to several fingertips at a range of statistical thresholds, and (iii) by computing an overlap ratio [Krause et al., 2001; Kurth et al., 2000]. All measures show that activation maps for adjacent fingertips show some degree of overlap in virtually all regions of S1. We found that a significant proportion of voxels in S1 responded to more than three adjacent fingertips (Fig. 4C). Given that cortical representations of nonadjacent fingertips are separated by at least 10 mm (Table 3), it is unlikely that this overlap can be explained by hemodynamic blurring of nonoverlapping cortical representations.

In a previous study we used a phase‐encoding design [Sanchez‐Panchuelo et al., 2010] to show somatotopically ordered fingertip‐specific cortical bands extending from the posterior bank of the central sulcus to the post‐central gyrus. The phase‐encoding design (and analysis) attributes a single stimulation location to each voxel, and is therefore not suited to revealing spatial overlap of BOLD responses (Dumoulin and Wandell, 2008].

In contrast to our findings here, other previous studies using block designs at high or ultra high‐field [Nelson and Chen, 2008; Schweizer et al., 2008; Stringer et al., 2011] have reported much less overlap. This discrepancy in observed overlap may be due to differences in statistical power, either because of lower SNR at 3T compared with 7T, particularly at higher spatial resolution [Nelson and Chen, 2008; Schweizer et al., 2008], or due to conservative multiple comparison procedures [Stringer et al., 2011]. Figure 4C illustrates that higher statistical thresholds (or equivalently, less statistical power at a given threshold) would result in the majority of voxels in both anterior and posterior ROIs showing activation to only one fingertip stimulation condition, leading to an underestimation of overlap. It is unlikely that the large overlap of responses seen in our results is due to inflated type 1 error rates in statistical analysis as we took care to eliminate sources of statistical bias by (i) accounting for the noise temporal correlation [Smith et al., 2007] and (ii) applying family‐wise error (FWE) adjusted thresholds at the single‐subject level. Further, although we aimed to increase the power of statistical tests by restricting the analysis to voxels in the vicinity of S1 using an independent localizer paradigm, it is likely that the overlap of responses may have been under‐estimated in our study. First, Bonferroni‐type methods, such as the Holm method used here, are inherently over‐conservative as they largely ignore spatial correlations between voxels. Second and more importantly, voxels that respond to the stimulation of all five fingertip locations will not completely return to baseline in the rest periods of the event‐related experiment, ultimately leading to an under‐estimation of the magnitude of parameter estimates.

A further reason as to why we found more overlap may be that we used relatively short stimulation durations in an event‐related design, while previous studies used block designs with long periods of stimulation. In support of this idea, a recent optical imaging study in squirrel monkeys showed that longer stimulation periods result in more focused activation in S1 [Simons et al., 2007]. Similarly, a recent fMRI study at 7T using a block design reported no overlap in the anterior part of S1 (identified as area 3b using probabilistic cytoarchitectonic maps), and less overlap than we report in posterior parts of S1 [Martuzzi et al., in press].

### Overlap Increases from the Anterior to Posterior Regions of S1

The different quantitative measures all indicate that the amount of overlap increased on moving from anterior to posterior in S1. In the anterior region (roughly corresponding to the posterior bank of the central sulcus) the overlap appears to be fairly limited, as reported previously [Schweizer et al., 2008; Stringer et al., 2011]. However, analyses based on ROI‐averaged magnitude estimates, as well as a previously proposed overlap ratio index, show that overlap in the anterior of S1 is significant at the group level, with some finger‐specific anterior ROIs responding to a maximum of three adjacent fingers.

In contrast, a large proportion of voxels in the posterior half of the ROIs (roughly corresponding to the post‐central gyrus) responded to the stimulation of multiple fingertips (up to five). As a result, somatotopic organization is not readily apparent in this region on superimposed individual fingertip activation maps. Our ROI analysis however shows that despite the large overlap, there is still a degree of specificity with ROIs responding maximally to the corresponding fingertip and decreasingly with distance from the preferred fingertip.

The spatial variation in overlap is consistent with previous group studies at lower‐field [Krause et al., 2001; Kurth et al., 2000; Ruben et al., 2006] and electrophysiological results in primates, provided that the spatial blurring of the hemodynamic responses is taken into account (see below).

We also note that the cortex representing the thumb appeared more selective than other fingers, in both the anterior and posterior parts of S1 (see Fig. 2B, C and 4B). Other fMRI studies have found that the representation of the thumb was relatively larger than that of other fingers in S1 [Martuzzi et al., in press] but, to our knowledge, have not reported such relative selectivity. Whether this is related to the relative independence of the thumb compared to other fingers will require further investigation.

### Activation Patterns and Relations to Cytoarchitectonic Subregions of S1

We did not see any clear indication of distinct clusters of activation corresponding to areas 3b, 1, and 2, as has been reported by others [Nelson and Chen, 2008; Stringer et al., 2011]. In our data, activation on the post‐central gyrus can be better described as a patchwork of clusters, some showing fingertip specificity in the anterior ROI of S1 and others showing significant activation in response to stimulation of any fingertip in the posterior ROI.

We defined this boundary between anterior and posterior regions based on functional criteria by assessing the change in overlap on the superimposed fingertip activation maps on the cortical surface. Therefore, this division was not anatomical and is not meant a priori to represent the boundary between cytoartchitectonically defined areas 3b and area 1. For all subjects, however, the boundary was found to lie close to the transition between the posterior bank of the central sulcus and the post‐central gyrus (although for subjects 1 and 6, it was clearly on the post‐central gyrus) and to follow the main direction of the central sulcus. This location and its inter‐subject variability are consistent with the location and variability of the 3b/1 border found in human post‐mortem cytoarchitectonic studies [Geyer et al., 1999].

Our results are therefore compatible with the hypothesis that voxels activated in the posterior bank of the central sulcus belong to area 3b, an area in which the cortical representation of adjacent fingertips shows little overlap in primates [Iwamura et al., 1983a; Pons et al., 1987]. Some overlap between responses to the stimulation of adjacent fingertips would be expected due to the spatial blurring introduced by the hemodynamic response, even if the underlying cortical representations did not overlap [Parkes et al., 2005; Shmuel et al., 2007], and this blurring could explain the limited overlap that we found in the posterior bank of the central sulcus. Assuming that the BOLD point‐spread function is spatially invariant, the increase in overlap in the post‐central gyrus would therefore reflect genuine overlapping receptive fields, compatible with larger receptive fields that encompass several adjacent fingertips in areas 1 and/or 2 [Iwamura et al., 1983b, 1985]. In several subjects, the ER data also showed overlapping BOLD responses in regions posterior to the post central gyrus and anterior to the posterior bank of the central sulcus. Even though they respond to fingertip stimulation, these regions are probably not somatotopically organized at the level of the fingertips since they did not generate ordered cortical bands in the phase‐encoding localizer. This finding is compatible with the less precise somatotopic maps previously found in areas 3a [Krubitzer et al., 2004] and in areas 2 [Pons et al., 1985] and 5 [Seelke et al., 2011]. Some subjects (e.g., subjects 1 and 4) also showed an apparently somatotopically organized representation of the fingertips in regions anterior to S1. Whereas we cannot exclude that somatotopic maps found in the anterior bank of the central sulcus (e.g., subject 4) are due to possible spatial spread of activation from S1 across the sulcus due to registration errors, somatotopic maps on the precentral gyrus (e.g., subject 1) could correspond to somatotopically organized somatotopic responses in the motor cortex (for a more detailed discusion, see Besle et al., in press). At this point, we cannot exclude the explanation that small movements of the fingers in reaction to the vibrotactile stimulation might be responsible for this anterior map [Olman et al., 2011].

Note that subregions of S1 were originally defined in the primate using functional criteria (a reversal of somatotopy between the proximal and distal parts of fingertips) and subsequently found to coincide with cytoarchitectonic areas [Merzenich et al., 1978]. Cytoarchitectonic studies on post‐mortem human brains do suggest that the same subregions of S1 can be found in humans as in monkeys [Geyer et al., 1999; Grefkes et al., 2001]. There have been some attempts using fMRI at 1.5 and 4T [e.g., Blankenburg et al., 2003; Overduin and Servos, 2004], to identify such subareas 3a, 3b, 1 and 2 by measuring the somatotopic organization along the proximal‐distal axis of a digit to define the expected reversals of these maps, and a recent study at 7T has demonstrated the functional parcellation of all sub‐areas [Sanchez‐Panchuelo et al., 2012]. Another way of delimiting the borders between subareas of S1 would be to use myelin‐sensitive high‐resolution structural MR sequences [Geyer et al., 2011], and we have recently published work in which we attempt to apply this approach [Sanchez‐Panchuelo et al., in press]. Combining such sub area maps with maps of spatial overlap of digits will allow confirmation of whether the degree of spatial overlap varies across sub areas 3a, 3b, 1, and 2.

A potentially complicating factor for the interpretation of the origin of spatial overlap is the difference in the distribution of large draining veins across sulci and gyri. Despite being insensitive to intravascular signals [Yacoub et al., 2001], gradient‐echo BOLD contrast at 7T is sensitive to extra‐vascular contributions from large veins [Duong et al., 2003]. As the change in fingertip specificity in most of our subjects occurs almost exactly in an area of transition between a sulcus and a gyrus, it could at least partly be explained if there was a higher density of large draining veins at the cortical surface in the post‐central gyrus compared with the central sulcus. However, there is currently no quantitative measure of such density [see Duvernoy, 1999 for a qualitative description]. Our examination of vein maps (see Fig. 5) showed that signals from large veins alone are unlikely to explain the pattern of responses we see. However, large veins are ubiquitous on the cortical surface, and their contributions need to be addressed fully. This could be achieved by a comparison of gradient‐echo and spin‐echo BOLD data, since spin echo measurements, although providing lower sensitivity than the gradient echo contrast used, dephase the contributions from larger veins [Duong et al., 2003].

## CONCLUSIONS

This study has shown the feasibility and power of using an event‐related design at 7T for robust mapping of the somatotopic representation of fingertips in individual subjects and also highlights the additional benefit of event related designs in allowing the assessment of the spatial overlap of responses. As a result of the improved SNR afforded by ultra‐high magnetic field, we have been able to show that cortical responses to different fingertips overlap in S1, that the overlap increases from anterior (posterior bank of the central sulcus) to posterior (post‐central gyrus) and that most of this overlap is not easily explained by the contributions of veins to hemodynamic blurring. The use of event‐related designs at 7T opens up the possibility of assessing more subtle transient changes in the responses of somatosensory cortex in individual subjects, potentially even for diagnostic purposes, such as those due to the effects of cognitive demand, attention, and so on or changes due to neural plasticity.
